# Feasibility of modified radical mastectomy with nipple-areola preservation combined with stage I prosthesis implantation using air cavity-free suspension hook in patients with breast cancer

**DOI:** 10.1186/s12957-021-02220-7

**Published:** 2021-04-10

**Authors:** Jiaqi Liu, Heshan Yu, Yuxiao He, Ting Yan, Yu Ding, Jun Chu, Ning Gao, Xiaona Lin, Yanbin Xu, Guijin He

**Affiliations:** 1 First Ward of Thyroid Breast Surgery, Central Hospital of Zibo, Zibo, China; 2 Department of Breast Surgery, Shengjing Hospital of China Medical University, Shenyang, China; 3 School of Medical Technology, Zibo Vocational Institute, Zibo, China; 4 Department of Second Breast Surgery, Shengjing Hospital of China Medical University, Shenyang, China

**Keywords:** Breast cancer, Modified radical mastectomy, Mammaplasty, Breast implant

## Abstract

**Background:**

Mastoscopic surgery is proven to have lower incidence of postoperative complications and better postoperative recovery than traditional breast cancer surgery. This study aimed to examine the feasibility of mastoscopic modified radical mastectomy (MRM) with skin nipple-areola preservation under air cavity-free suspension hook and stage I silicone prosthesis implantation (SMALND) compared with routine MRM.

**Methods:**

This was a retrospective study of patients who underwent MRM for breast cancer at the Shengjing Hospital Affiliated to China Medical University between January 1, 2019, and June 30, 2019. Surgical outcomes, complications, satisfaction, and quality of life (Functional Assessment of Cancer Therapy-Breast [FACT-B] [Chinese version]) were compared between the two groups.

**Results:**

A total of 87 patients were enrolled, with 30 underwent SMALND and 57 underwent routine MRM. The intraoperative blood loss in the SMALND group was lower than in the control group (165.3±44.1 vs. 201.4±52.7 ml, *P*=0.001), the operation time was longer (220.5±23.9 vs. 155.6±9.2 min, *P*<0.001), daily axillary drainage volume was smaller (20.2±3.6 vs. 24.1±3.0 ml, *P*<0.001), daily subcutaneous drainage volume was smaller (15.5±2.3 vs. 19.3±3.5 ml, *P*<0.001), the discharge time was shorter (7.5±1.6 vs. 9.0±1.8 days, *P*<0.001), and FACT-B scores were higher (83.8±5.6 vs. 72.1±4.6, *P*<0.001). The overall satisfaction was higher in the SMALND group than in the controls (76.7% vs. 54.4%, *P*=0.041). Compared with the controls, the occurrence rates of nipple and flap necrosis, upper limb edema, and paraesthesia in the SMALND group were lower within 6 months (all *P*<0.05).

**Conclusions:**

Compared with traditional MRM, SMALND had better surgical outcomes, higher satisfaction, higher quality of life, and lower complication rates.

## Background

Although it is not an essential organ, the breast is important for women’s appearance, quality of life, and psychological health [1, 2]. With the precise treatment of breast cancer, these patients’ lifespan is now significantly longer than a few decades ago [3]. Still, how to improve the patients’ quality of life after treatments is now a significant challenge [3]. Breast preservation can significantly improve the quality of life of women after breast surgery [4–7].

Nevertheless, breast-conserving surgery (BCS) has some limitations in clinical practice, and the need for radiotherapy after surgery causes many patients to lose their motivation for BCS [8, 9]. On the other hand, traditional modified radical mastectomy (MRM) significantly increases the occurrence of complications such as upper limb edema and paraesthesia, and the surgical scar from on the chest and the axilla will affect the esthetics and limit the movement of the shoulder joint to some extent [10].

Mastoscopic surgery is becoming popular. The literature confirmed that the complications and postoperative recovery of patients with mastoscopic surgery were significantly lower than those of traditional breast cancer surgery patients [11–13]. Based on this, we propose a procedure of mastoscopic MRM with nipple-areola preservation combined with stage I prosthesis implantation. This new procedure could be proposed to patients who are not able or willing to undergo BCS and could also circumvent the shortcomings of mastoscopic surgery, such as insufficient armpit space and unstable pneumoperitoneum. It allows retaining the breasts after MRM and ensures a good shape after surgery and solves the problems of unstable and narrow axillary space during surgery.

Therefore, the present study aimed to examine the feasibility of mastoscopic MRM with skin nipple-areola preservation under air cavity-free suspension hook and stage I silicone prosthesis implantation compared with routine MRM.

## Methods

### Study design and patients

This study was a retrospective study of 87 patients who underwent MRM for breast cancer at the Second Breast Surgery of Shengjing Hospital Affiliated to China Medical University between January 1, 2019, and June 30, 2019. This study was approved by the ethics committee of Shengjing Hospital Affiliated to China Medical University [2017PS009J]. The patients signed an informed consent form for their data to be anonymously used for research purposes.

The inclusion criteria were 1) breast cancer at clinical stage I and II, no noticeable skin and deep infiltration [10], 2) N0 or N1 disease by clinical examination, ultrasound, mammography, and magnetic resonance imaging (MRI) [10], 3) refused BCS or had contraindications to BCS, 4) indications for mastoscopic MRM with skin nipple-areola preservation combined with stage I prosthesis implantation under air cavity-free suspension hook (patients with indications for conventional axillary lymph node dissection (ALND), no history of axillary surgery, clinical examination, ultrasound, mammography, and magnetic resonance imaging (MRI) showing N0-N1, and swollen lymph nodes had no adhesion with blood vessels and nerves), and 5) intraoperative frozen section of the areola and glands under the areola indicated no cancer cell infiltration.

The exclusion criteria were 1) combined with severe chronic or disabling diseases such as hypertension, diabetes, etc., 2) intellectual or mental factors limiting communication, 3) stage III-IV breast cancer [10], or 4) could not meet the indications of MRM with skin nipple-areola preservation combined with stage I prosthesis implantation under air cavity-free suspension hook.

### Grouping

During the study period, 30 patients underwent mastoscopic modified radical mastectomy with skin nipple-areola preservation under air cavity-free suspension hook, and stage I silicone prosthesis implantation (SMALND group), and 57 patients with breast cancer underwent MRM (control group). When a patient was eligible for SMALND, the advantages and disadvantages of SMALND were explained to the patient. Then, the patient was free to select the procedure they preferred.

The advantages are that 1) the breast shape is preserved after surgery, 2) patients with mastoscopic surgery experience less trauma and have a faster postoperative recovery, shorter hospital stay, and lower occurrence rate of postoperative complications, 3) the therapeutic effect is not significantly different from that of MRM, and 4) it avoids the psychological regret of patients who had lost the opportunity of breast plastic surgery after MRM.

The disadvantages are that 1) it is more expensive (10,000-20,000 yuan higher than MRM), 2) there may be asymmetry of the breasts and a difference in touch feeling, and 3) if the prosthesis is infected and damaged, it will have to be removed.

### SMALND group

**Fig. 1:**
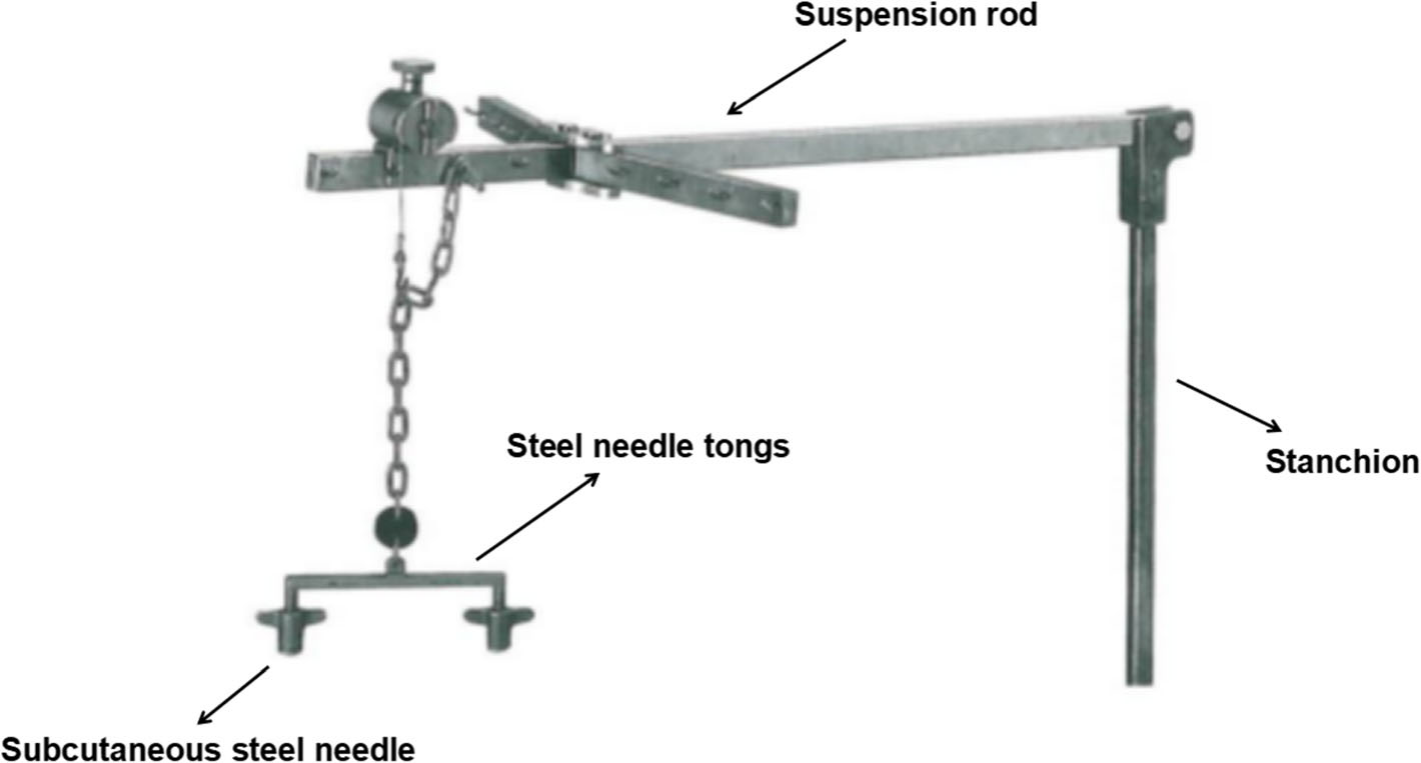
The pneumoperitoneum-free suspension instrument

**Fig. 2:**
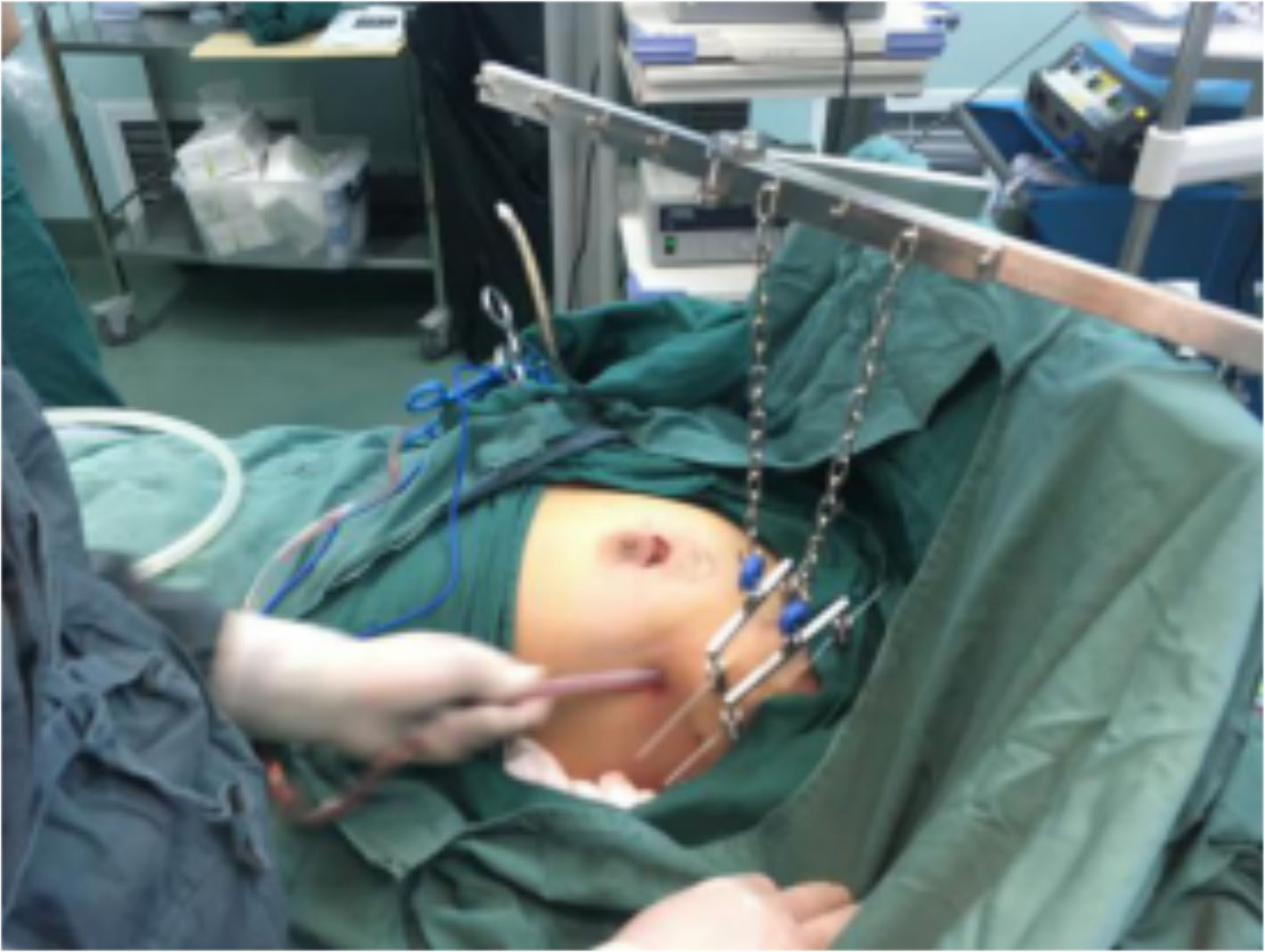
Liposuction under traction with suspension hook

**Fig. 3:**
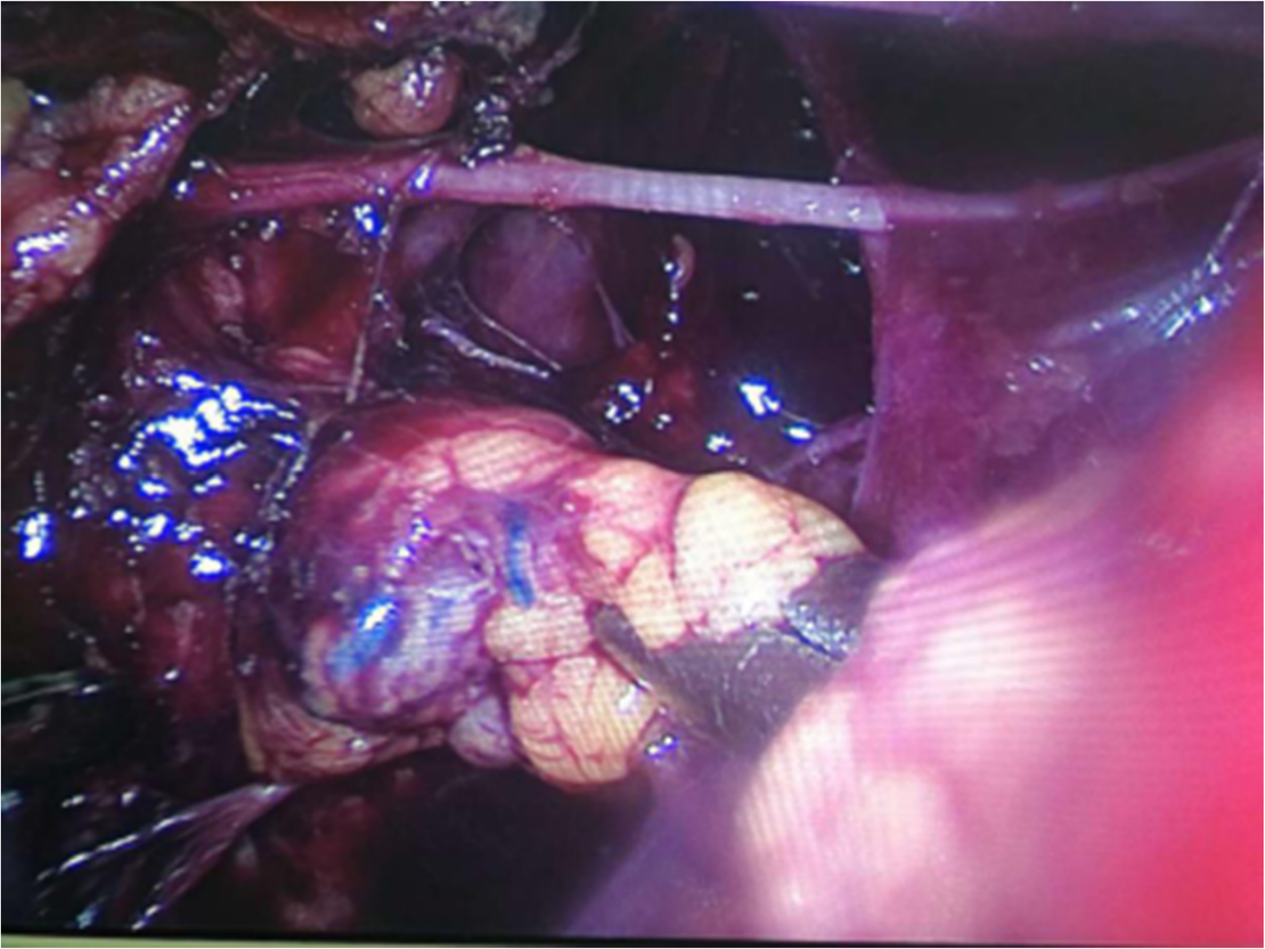
Amplified vessels and sentinel lymph nodes under laparoscopy

**Fig. 4:**
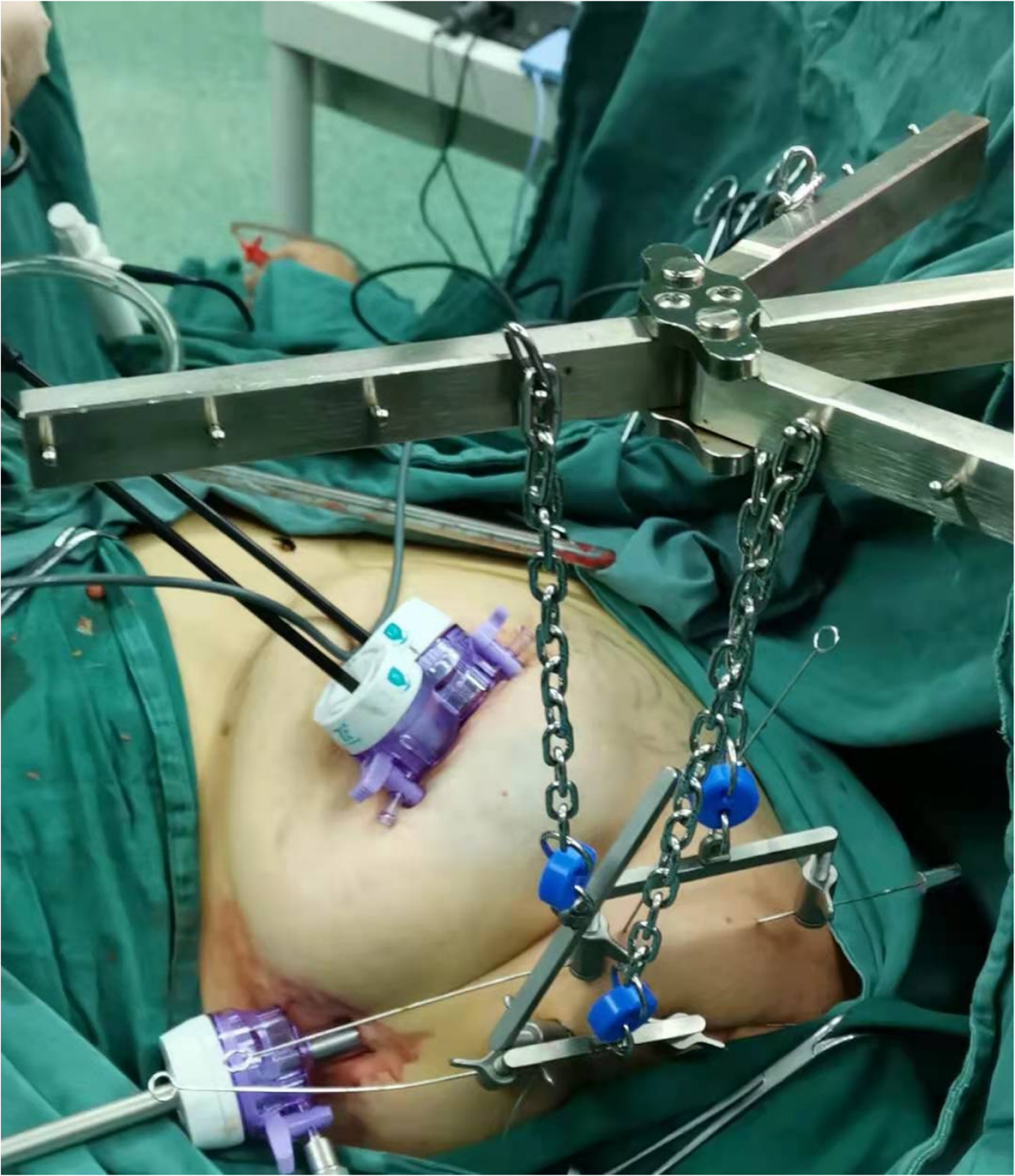
Mastoscopic breast glandectomy under the suspension hook

All patients were operated on by the same surgical team, led by the chief of the Department of Second Breast Surgery, Shengjing Hospital, affiliated to China Medical University (postdoctoral graduate, >20 years of experience). The patient was placed supine. The upper limb of the affected side was abducted. The breast tumor resection was performed first. The specimen was sent for intraoperative pathology. When confirmed positive, the glands under the nipple and areola were taken from the pathological incision, and an intraoperative frozen section was performed. When confirmed negative, a carnarin suspension injection was performed intradermally with 0.5 ml at multiple points around the areola. The lipolytic solvent was formulated with 125 ml of 0.9% sodium chloride solution, 125 ml of distilled water, 20 ml of lidocaine, and 0.5 ml of adrenaline hydrochloride. The lipolytic solvents were injected subcutaneously at multiple points in the armpit. The patient was massaged at the injection sites, and the solvent was left to react for 20 min. A pneumoperitoneum-free suspension device (Fig. 1) was installed, and the axillary skin was pulled up. A 10-mm trocar was then inserted into the anterior axillary incision at the lower edge of the breast and subcutaneously punctured. After puncturing the axillary site, an aspirator was used to mash and suction the fatty tissues (Fig. 2). The endoscope was inserted to explore the axilla, and 5- and 10-mm trocars were used for a subcutaneous puncture at the incision of the midline of the axillary and the incision of the original mass under mastoscopic monitoring. After a successful puncture, separation forceps and grasping forceps were placed in the trocars. An electric hook was used to cut off the blood vessel branches. Sentinel axillary lymph nodes were dissected using methylene blue (Fig. 3) [12–14], and a frozen section was examined during the operation. If the intraoperative frozen section indicated lymph node metastasis, further mastoscopic (Olympus, Shenzhen, China) lower ALND was performed. The pneumoperitoneum-free suspension device (Daoke Medical Group, Shanghai, China) was installed on the breast skin. The breast glands were then removed under mastoscopy with separation forceps and grasping forceps (Fig. 4) and removed from the pathological incision.

**Fig. 5:**
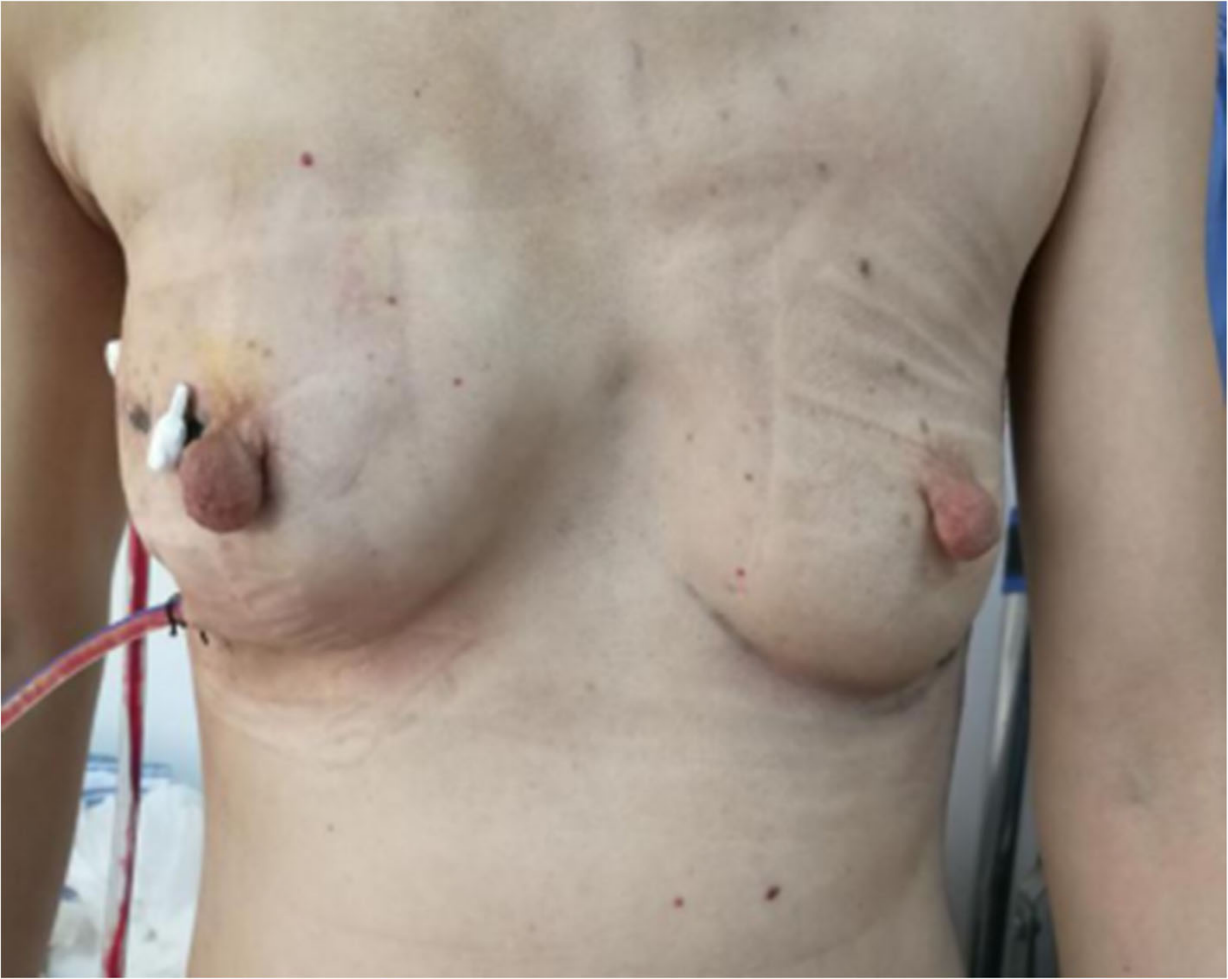
Two 470 drainage tubes were placed through the trocar port of the mastoscope for 3 days after surgery

5-FU (1000 mg) was added to distilled water. The pectoralis major and pectoralis minor muscles were separated from the pathological incision, and the prosthesis was implanted. If the sentinel lymph nodes were negative, a silicone prosthesis (Haiweining Plastic Products Co., Ltd., Shanghai, China) was implanted during stage I. If the sentinel lymph nodes were positive, a dilator was placed, and the silicone prosthesis was implanted 6 months after the end of the radiotherapy and chemotherapy. The drainage tube was placed, and the pectoralis major and pectoralis minor fascia were sutured. Subcutaneous drainage tubes were placed at the axilla and costal arch (Fig. 5). Titanium nickel wire was used for the continuous subcutaneous suture of the incision. An elastic bandage was used for bandaging.

### Control group

MRM was performed as in the original NSABP B-06 trial [15]. After successful general anesthesia, the patient was placed in the supine position, and the upper limb of the affected side was abducted. After resection, the tumor was sent for rapid pathological examination during the surgery. If malignancy was confirmed, methylene blue injection was injected at multiple areola points, and MRM was performed. A Stewart incision on the affected side was made. The flaps were dissociated, up to the collarbone, down to the costal arch, inward to the midline, and out to the midaxillary line. The breast was removed from the pectoralis major muscle. SLNB was performed using methylene blue. If lymph node metastasis was observed during the intraoperative frozen section, the first, second, and third levels and intermuscular lymphatic adipose tissues were dissected. The axillary lymph nodes, usually marked by the pectoralis minor muscle, were divided into three levels. The first level was located on the lateral side of the pectoralis minor muscle. Besides, the lymph nodes between the pectoralis minor and major muscles were also included in this level. The second level referred to the axillary vein lymph nodes deep in the pectoralis minor muscle. The third level was the lymph nodes in the subclavian vein inside the pectoralis minor. The long thoracic nerve and thoracodorsal nerve were retained. 5-FU (1000 mg) was added to distilled water. Subcutaneous drainage tubes were placed at the axilla and costal arch. An inventory was made for gauze and instruments to make sure that the number was correct. Titanium nickel wire was used for the suture of the incision. An elastic bandage was used for bandaging.

### Outcomes

The operation time, intraoperative blood loss, postoperative daily subcutaneous and axillary drainage, postoperative subcutaneous and axillary extubation time, the occurrence of postoperative nipple and flap necrosis, and the degree of satisfaction were recorded routinely. Upper limb edema and paraesthesia at 1, 3, and 6 months after surgery were assessed in the two groups. The grading of pain was based on NCCN pain guidelines [16]. Patients’ satisfaction was evaluated routinely at 6 months postoperatively. The satisfaction was reported by the patients and graded as satisfied, moderately satisfied, not bad, and not satisfied. The overall satisfaction was defined as the proportion of satisfied and moderately satisfied patients. The breast shape of the patients who underwent SMALND was evaluated by an orthopedist using the Harris scale at 6 months postoperatively [17]. Breast shape satisfaction was defined as the proportion of excellent and good grades. Patient quality of life was routinely assessed using the Functional Assessment of Cancer Therapy-Breast (FACT-B) (Chinese version) [18].

### Statistical analysis

All data were analyzed using SPSS 25.0 (IBM, Armonk, NY, USA). The normal distribution of continuous data was tested using the Kolmogorov-Smirnov test. The continuous data with a normal distribution are presented as means ± standard deviations and were analyzed using the independent sample *t* test. Categorical data are presented as numbers (percentages) and were analyzed using the chi-square test. The differences were considered statistically significant when *P*<0.05.

## Results

### Characteristics of the patients

A total of 87 patients were enrolled, with 30 underwent SMALND and 57 underwent routine MRM. There were no differences between the two groups regarding the clinical and pathological characteristics of the patients and cancers (all *P*>0.05) (Table 1).

**Table 1:** Characteristics of the patients

Characteristics	SMALND (*n*=30)	Control (*n*=57)	*P*
Age, years, mean±SD	43.7±7.7	45.2±8.4	0.390
BMI, kg/m^2^, mean±SD	22.2±2.0	21.8±2.7	0.477
Stage, *n* (%)			0.555
I	17 (56.7)	36 (63.2)	
II	13 (43.3)	21 (36.8)	
Height, m, mean±SD	1.63±0.04	1.63±0.04	0.606
Weight, kg, mean±SD	58.97±6.30	58.02±6.01	0.500
Subtypes, *n* (%)			
Luminal A	4 (13.3)	8 (14.0)	0.928
Luminal B (HER−)	6 (20.0)	14 (24.6%)	0.631
Luminal B (HER+)	6 (20.0)	16 (28.1)	0.410
HER2+	9 (30.0)	8 (14.0%)	0.074
Triple negative	5 (16.7)	11 (19.3)	0.873
ER-positive, *n* (%)	16 (53.3)	38 (66.7)	0.223
PR-positive, *n* (%)	16 (53.3)	38 (66.7)	0.223
HER2-positive, *n* (%)	15 (50.0)	24 (42.1)	0.482

*SD* Standard deviation, *BMI* Body mass index, *SMALND* Mastoscopic modified radical mastectomy with nipple-areola preservation combined with stage I prosthesis implantation under air cavity-free suspension hook, *ER* Estrogen receptors, *PR* Progesterone receptor

### Surgical characteristics

The operation time of the SMALND group was significantly longer than that of the control group (220.5±23.9 vs. 155.6±9.2, *P*<0.001). Still, the intraoperative blood loss, the average daily subcutaneous and axillary drainage volume, and the average subcutaneous and axillary extubation time were smaller than in the control group (all *P*≤0.001) (Table 2). The number of sentinel lymph nodes was significantly higher in the SMALND group than in the control group (5.9±1.0 vs. 7.2±2.0, *P*<0.001, Table 2). In the SMALND group, 21 patients underwent SLNB, and nine suggesting axillary lymph node metastasis underwent ALND after SLNB. In the control groups, 44 patients underwent SLNB, and 13 patients underwent ALND after SLNB. There was no significant difference in axillary lymph nodes between the two groups (*P*=0.463) (Table 2).

**Table 2:** Surgical characteristics

Surgical characteristics	SMALND (*n*=30)	Control (*n*=57)	*P*
Intraoperative blood loss, ml, mean±SD	165.3±44.1	201.4±52.7	0.001
Operation time, min, mean±SD	220.5±23.9	155.6±9.2	<0.001
Number of sentinel lymph nodes, mean±SD	5.87±1.04	7.16±2.04	<0.001
Treatment of lymph nodes, *n* (%)			0.463
SLNB	21 (70.0)	44 (77.2)	
ALND	9 (30.0)	13 (22.8)	
Average daily subcutaneous drainage, ml, mean±SD	15.5±2.3	19.3±3.5	<0.001
Average daily axillary drainage, ml, mean±SD	20.2±3.6	24.1±3.0	<0.001
Subcutaneous extubation time, days, mean±SD	6.2±1.1	9.0±1.8	<0.001
Axillary extubation time, days, mean±SD	7.5±1.6	9.0±1.8	<0.001
Average discharge time, days, mean±SD	7.5±1.6	9.0±1.8	<0.001

*SD* Standard deviation, *SMALND* Mastoscopic modified radical mastectomy with nipple-areola preservation combined with stage I prosthesis implantation under air cavity-free suspension hook, *SLNB* Sentinel lymph node biopsy, *ALND* Axillary lymph node dissection

### Postoperative outcomes

In the SMALND group, 14 patients were satisfied, and nine were moderately satisfied, for overall satisfaction of 76.7%. Seven patients were satisfied in the control group, 24 were moderately satisfied, for overall satisfaction of 54.4%. The overall satisfaction was higher in the SMALND group than in the controls (76.7% vs. 54.4%, *P*=0.041) (Table 3). There were 12 patients with excellent breast shape and 14 with good shape, leading to a breast shape satisfaction rate of 86.7%. In the FACT-B, compared with the control group, the patients in the SMALND group reported higher scores of social-family well-being (19.8±2.2 vs. 15.2±1.6, *P*<0.001), functional well-being (19.3±1.7 vs. 16.3±1.7, *P*<0.001), additional concerns (18.4±1.6 vs. 14.9±1.6, *P*<0.001), and total FACT-B (83.8±5.6 vs. 72.1±4.6, *P*<0.001).

**Table 3:** Postoperative outcomes

Postoperative outcomes	SMALND (*n*=30)	Control (*n*=57)	*P*
Patients’ overall satisfaction, *n* (%)	23 (76.7)	31 (54.4)	0.041
FACT-B score, mean±SD			
Physical well-being	13.7±2.9	13.3±2.7	0.515
Social-family well-being	19.8±2.2	15.2±1.6	<0.001
Emotional well-being	12.5±1.8	12.3±1.3	0.632
Functional well-being	19.3±1.7	16.3±1.7	<0.001
Additional concerns	18.4±1.6	14.9±1.6	<0.001
Total scores	83.8±5.6	72.1±4.6	<0.001
Breast shape satisfaction, *n* (%)	26 (86.7)		
Complications, *n* (%)			
Nipple and skin flap necrosis	0	7 (12.3)	0.045
Upper limb edema			
1 month postoperative	4 (13.3)	19 (33.3)	0.044
3 months postoperative	1 (3.3)	11 (19.2)	0.040
6 months postoperative	0	7 (12.3)	0.045
Paraesthesia			
1 month postoperative	7 (23.3)	26 (45.6)	0.044
3 months postoperative	3 (10.0)	15 (26.3)	0.027
6 months postoperative	0	9 (15.8)	0.031

*SD* Standard deviation, *SMALND* Mastoscopic modified radical mastectomy with nipple-areola preservation combined with stage I prosthesis implantation under air cavity-free suspension hook, *FACT-B* Functional Assessment of Cancer Therapy-Breast

### Postoperative complications

The postoperative occurrence rate of nipple and skin flap necrosis in the SMALND group was 0% (0/30), which was significantly lower than the 12.3% (7/57) in the control group (*P*=0.045).

The occurrence rates of upper limb edema in the SMALND group at 1 month, 3 months, and 6 months postoperatively was significantly lower than control group (all *P*<0.05, Table 3). At 1 month, in the four patients in the SMALND group who developed upper limb edema, edema was mild in three patients, and their function was not affected, but the fourth patient had some function limitation. Among the 19 patients in the control group who developed upper limb edema, 15 had mild upper limb edema, and four had some function limitation. At 3 months, one patient in the SMALND group had mild upper limb edema, while 11 patients in the control group had upper limb edema (10 mild cases, and one with function limitation). At 6 months, there were no patients with upper limb edema in the SMALND group, while seven patients in the control group had upper limb edema, including six mild cases.

The occurrence rates of paraesthesia in the SMALND group at 1 month, 3 months, and 6 months postoperatively was significantly lower than control group (all *P*<0.05, Table 3). At 1 month, seven patients in the SMALND group had paraesthesia, including five cases of pain grade I-II, four with hypoesthesia or numbness, and two of soreness or heaviness; 26 patients in the control group had paraesthesia, including two cases of pain grade I-II, 23 of hypoesthesia or numbness, and one of soreness or heaviness. At 3 months, three patients in the SMALND group had paraesthesia, including one of pain grade I-II, and two hypoesthesia or numbness; 15 patients in the control group had paraesthesia, including one case of pain grade I-II, and 14 cases of hypoesthesia or numbness. At 6 months, no patient in the SMALND group had paraesthesia; six patients in the control group had paraesthesia, including nine hypoesthesia cases or numbness.

## Discussion

Traditional MRM is associated with upper limb edema, paraesthesia, and a scar that may limit shoulder movement. Therefore, this study aimed to examine the feasibility of mastoscopic MRM with skin nipple-areola preservation under air cavity-free suspension hook and stage I silicone prosthesis implantation compared with routine MRM. The results suggest that compared with traditional MRM, mastoscopic MRM with skin nipple-areola preservation under air cavity-free suspension hook and stage I silicone prosthesis implantation had better surgical outcomes, satisfaction, and quality of life and lower complication rates. The shape of the breast was reconstructed based on preserving the patient’s skin and nipple-areola, improving the patient’s quality of life and is worth clinical promotion.

Compared with the 57 patients who underwent conventional ALND, the 30 patients who underwent mastoscopic MRM with skin nipple-areola preservation combined with stage I prosthesis implantation under air cavity-free suspension hook had smaller intraoperative blood loss, smaller postoperative drainage, shorter extubation time, faster postoperative recovery, and a lower occurrence rate of postoperative complications. It helped women retain their breasts and led to good cosmetic results. Mastoscopic ALND and glandular resection could be completed with only two 10-mm trocar ports. Lymph nodes could be removed through the trocar, and the glands could be removed entirely. After surgery, the subcutaneous and axillary drainage tubes could also be placed through the two trocar holes. Second, compared with MRM, ALND under suspension mastoscopy significantly reduced the postoperative occurrence rates of nipple and flap necrosis, upper limb edema, and paraesthesia. Mastoscopic glandectomy and ALND were performed by lipolysis to establish a cavity into the subcutaneous breasts and axilla, which did not require electrosurgical dissociation, led to little damage, and had almost no effect on the flap, thereby significantly reducing the postoperative occurrence of flap necrosis. Mastoscopic ALND can magnify the axillary nerves, blood vessels, and lymph vessels by 8-10 times. Lymph nodes, axillary veins, thoracodorsal nerve and blood vessels, intercostal wall nerves, and lymph vessels could be observed under the microscope. During surgery, the intercostal wall nerves could be retained to the greatest extent, and the occurrence of paraesthesia could be significantly reduced. The magnifying effect of mastoscopy could be finely manipulated to avoid damage to blood vessels and lymphatic vessels and reduce the occurrence of postoperative upper limb edema. Compared with MRM, patients with mastoscopic glandectomy and ALND had smaller intraoperative blood loss, faster postoperative recovery, and shorter extubation time. Under the effect of microscopic magnification and through the delicate operation, mastoscopic glandectomy and ALND avoided damage to most blood vessels, which could significantly reduce the amount of intraoperative and postoperative blood loss and shorten the extubation time and reduce the length of hospital stays of patients. The most crucial point was that surgery removed the tumor and helped the patients retain their breasts, which would significantly improve their quality of life and body image.

Those good outcomes are comparable to those achieved using classical mastoscopic surgery [11, 12, 14, 19, 20], Ding et al. [12] randomized 60 patients to mastoscopic SLNB, conventional SLNB, and SLNB with lipolysis and showed that mastoscopic SLNB performed at least as well than conventional SLNB. Luo et al. [11]e compared 500 patients with MRM and 496 patients who underwent mastoscopy and showed that mastoscopy had advantages in terms of healing, complications, function preservation, and cosmetics. These results are supported by other studies [14, 19, 20]. Mastoscopic glandectomy shares some principles with skin-sparing mastectomy, which is associated with good esthetic and oncological outcomes [21, 22]. Nevertheless, a true skin-sparing mastectomy is more invasive than mastoscopic surgery, and future studies should compare the two procedures in terms of complications and recovery.

During mastoscopic surgery, the establishment of the operative space is one of the key points to success. The pneumoperitoneum-free suspension instrument was first introduced by Nagai Hideo for the application of abdominal wall suspension laparoscopic cholecystectomy [23]. At present, there are no reports of pneumoperitoneum-free suspension instruments for axillary tissues. When comparing suspension surgery and traditional air cavity surgery [10, 11], excessive CO_2_ gas injection could adversely affect the patient’s visceral organs’ physiological functions and cause thermal injury to organs. In particular, in elderly patients with comorbidities, it increases the occurrence of certain unique complications, such as subcutaneous emphysema, gas embolism, hypercapnia, venous return block, shoulder pain, and nausea and vomiting [11]. The use of the suspension hook is the innovation of this study since there is no need for CO_2_ insufflation, avoiding the risks associated with it, including CO_2_ embolism and microembolism [24]. Although CO_2_ embolisms are rarely clinically significant, microembolisms can contribute to delayed healing and necrosis [24]. The air cavity-free suspension hook can not only avoid the abovementioned adverse reactions but also overcome the problems of air cavity instability and narrow axillary space due to gas leakage during ALND under endoscopy, which avoided the problems of blurring lens in the narrow space due to bleeding of small blood vessels and fogs produced by electric hook hemostasis. It improved the operation speed and shortened the operation time. Besides, the use of the pneumoperitoneum-free suspension instruments did not leave scars, was economical and cheap, and improved patient satisfaction. Still, this study did not compare mastoscopic glandectomy with CO_2_ insufflation vs. suspension hook. Future studies will have to examine that. In addition, all published studies of mastoscopy are limited to China. International studies will be necessary to confirm the generalizability of the results and confirm the oncological safety of the procedure.

The nipple-areolar complex (NAC) is an integral part of the female mammary gland. Preserving the NAC during breast reconstruction surgery of patients with breast cancer would significantly improve the cosmetic effect of the reconstructed breasts and improve the patients’ quality of life. In 1991, Toth and Lappert first proposed the concept of mammectomy with skin nipple-areola preservation, and it was later showed that the clinical effect was similar to that of MRM [5]. Preserving the skin, nipple, and areola might increase the possibility of residual cancer tissues and increase the risk of recurrence, which has been a concern of researchers. A recent study showed that MRM with skin nipple-areola preservation combined with stage I prosthesis reconstruction was a safe and effective surgical method and would not increase the local recurrence rate or distant metastasis rate [7]. Local recurrence of breast cancer is mainly derived from the mammary gland’s residual ductal epithelium, not the skin tissues of the breast [6]. Wijayanayagam et al. [25] reported that the prognosis of MRM with skin nipple-areola preservation was similar to that of MRM. Local recurrence was related to tumor size, staging, lymph node status, and degree of differentiation and had nothing to do with skin and nipple-areola preservation. Abdalla et al. [26] reported the safety of breast cancer surgery with skin and nipple-areola preservation. During MRM with skin and nipple-areola preservation, the glands under the nipple and areola should be taken for intraoperative frozen sections. Besides, SLNB was performed, and ALND was performed if the sentinel lymph nodes were positive.

The main complications after MRM with skin and nipple-areola preservation for breast cancer were nipple and flap necrosis, upper limb edema, and paraesthesia. These complications seriously affect the quality of life of patients after surgery. The literature on mastoscopic ALND confirmed that mastoscopic ALND had a faster recovery and a lower occurrence rate of complications than conventional ALND and could significantly improve the patients’ quality of life [12, 27, 28].

Conventional MRM requires skin flaps. An excessive electrosurgical operation can cause local skin burns. At the same time, it would cause excessive contraction or necrosis of the subcutaneous capillaries, which would affect the blood supply of the flaps after surgery. Improper preoperative incision design and excessive intraoperative tension of the suture could also cause ischemic necrosis of the local flap, which significantly prolongs the patient’s postoperative healing time. Axillary effusion or infection can obstruct lymphatic reflux, and fibrotic scars formed by excessive dissection hindered collateral circulation. All these factors can cause upper limb lymphedema and seriously affect the quality of life of the patients. The preservation of the intercostobrachial nerve can significantly reduce the occurrence rate of postoperative paraesthesia [29]. During conventional ALND, the intercostobrachial nerve is often cut due to excessive dissection or improper operation, which causes unusual or abnormal feelings such as postoperative upper limb pain, numbness, soreness and swelling, and other abnormal feelings. Excessive dissociated flaps, mammary glands, and axillary fat lymphoid tissues in conventional MRM would make the wound area larger, increase the postoperative blood loss and the average daily drainage volume, and relatively prolong the extubation time.

With the development of plastic surgery technology, the improvement of patients’ economic strength, and a better understanding of breast cancer, the purpose of treatment for breast cancer gradually changed from simply improving survival to improving survival and the quality of life. Breast reconstruction immediately after breast cancer is now a part of breast cancer’s local treatment, which is being accepted by more and more patients [30–32]. Compared with MRM, patients with mastoscopic MRM with skin nipple-areola preservation combined with stage I prosthesis implantation under air cavity-free suspension hook not only had a beautiful appearance but also had significantly higher satisfaction than that of the classical MRM in the control group. This new method is of great significance for patients to improve their perception of their body image, actively integrate into the social population after treatment, reduce the stigmata of the disease, and improve quality of life, as supported by the higher FACT-B scores and specific subscores of social family well-being, functional well-being, and additional concerns, and as supported by previous studies of breast reconstruction [33–37]. Such psychosocial benefits are seen with other types of reconstruction, such as nipple-sparing mastectomy with reconstruction using a deep inferior epigastric perforator flap [33], nipple-areola repositioning, implant, and modified inferior dermal flap [35], and nipple-sparing mastectomy [37]. Nevertheless, the possible advantage of the mastoscopic MRM with skin nipple-areola preservation combined with stage I prosthesis implantation under an air cavity-free suspension hook is that no flap is used, avoiding the morbidity of the donor area. Nevertheless, future studies should directly compare those different reconstruction methods within the same study.

This study has limitations. The sample size was small. The follow-up was short, preventing the determination of the recurrence rates. Because this was a retrospective study, quality of life was not formally assessed using validated questionnaires. Besides, the data of breast volumes of the patients were not routinely collected, which limited the analysis.

## Conclusions

In conclusion, mastoscopic MRM with skin nipple-areola preservation combined with stage I prosthesis implantation under an air cavity-free suspension hook is a feasible surgical method, which not only can improve patients’ satisfaction, satisfy the patients’ requirements for body image but also can reduce the occurrence of postoperative complications and improve the breast shape and the patients’ quality of life after surgery, as shown by the higher FACT-B scores. In cases where the breast cannot be preserved or there are concerns about the preservation of breast glands, but simultaneously caring about the breast’s shape, mastoscopic MRM with skin nipple-areola preservation combined with stage I prosthesis implantation under an air cavity-free suspension hook can be performed. This method is worth promoting and referencing for clinical doctors.

## Data Availability

The datasets used and/or analyzed during the current study are available from the corresponding author on reasonable request.

## Abbreviations

MRM: Modified radical mastectomy
BCS: Breast-conserving surgery
ALND: Axillary lymph node dissection
SMALND: Suspended axillary lymph node dissection
SLNB: Sentinel lymph node biopsy
